# Supplementary material for: Causal Associations of Circulating Lipids with Osteoarthritis: A Bidirectional Mendelian Randomization Study
Source: Nutrients. 2022 Mar 22;14(7):1327. doi: 10.3390/nu14071327 (PMC9000847; doi:10.3390/nu14071327)
Supplement: Supplementary file 1 [file nutrients-14-01327-s001.zip › nutrients-1640676-supplementary.pdf]

Supplemental materials

Causal Associations of Circulating Lipids with Osteoarthritis: A Bidirectional Two-Sample Mendelian Randomization Study

Contents

Supplemental Text S1. Mendelian randomization analysis of osteoarthritis (OA) on lipids………………………………………… ………..2

Supplemental Table S1. Associations of genetically predicted Apolipoprotein A1 (APOA1) with OA risks in MR analyses………….. ………3

Supplemental Table S2. Associations of genetically predicted HDL with OA risks in MR analyses……………………………………………4

Supplemental Table S3. Associations of genetically predicted TG with OA risks in MR analyses………………………………………………5

Supplemental Table S4. Associations of genetically predicted OA with lipids in MR analyses…………………………………………………6

Supplemental Figure S1. Diagram of the Mendelian randomization framework in this study……………………………………………………8

Supplemental Figure S2. Forest plots of variant specific inverse variance estimates for the causal association between lipids and OA……..……9

Supplemental Figure S3. Funnel plots of the causal association between lipids and OA……………………………………….…………………14

Supplemental Figure S4. Leave-one-out plots for assessing if the association between lipids and OA is driven by a single variant……..………19

Supplemental Text S1. Mendelian randomization analysis of osteoarthritis on lipids

Mendelian randomization (MR) uses genetic variants as instrumental variables (IVs) to determine if a potential intervention approach causally affects disease outcomes. MR analysis can mimic the effects of randomized control trials and avoid the limitations of observational studies because germline mutation is unaffected by environmental or lifestyle confounders. Furthermore, MR does not require individual-level data and can be implemented using summary statistics from genome-wide association studies (GWAS; also called "two-sample MR"). There are three basic assumptions for the MR analysis: 1) genetic variants can predict exposure (p < 5E-8), 2) genetic variants are not associated with any confounder (LD threshold: $r^2 < 0.001$), and 3) the association between genetic variants and outcomes is completely due to exposure.

The genetic IVs for knee osteoarthritis (KOA), hip osteoarthritis (HOA), and osteoarthritis of the knee or hip (KHOA) based on summary-level data from the UK Biobank (UKBB) were 10, 27, and 26.

We used the multiplicative random-effects inverse-variance weighted (IVW) model as the main statistical method. The main sensitivity analysis was based on the weighted median and the MR-Egger regression. The weighted median estimator was consistent even when up to 50% of the SNPs were invalid IVs, whereas the MR-Egger regression was performed by simply adding an intercept term of pleiotropy into the regression model for IVW estimation.

Supplemental Table S1 Associations of genetically predicted APOA1 with OA risks in MR analyses

Main outcome	Method	No. of SNPs	OR (95% CI)	*P* for association	*P* for heterogeneity test	*P* for MR-Egger intercept	*P* for MR-PRESSO Global test	Statistical power
KHOA	IVW	280	0.973(0.922-1.026)	0.315	1.45E-23	0.56		1.00
	MR-Egger	280	0.993(0.911-1.082)	0.872	1.21E-23			
	Weighted median	280	0.931(0.870-0.996)	0.039				
	MR-PRESSO (raw,5outliers)	275	0.982(0.979-0.985)	0.482			<1E-04	
KOA	IVW	279	0.962(0.902-1.026)	0.234	1.00E-21	0.30		0.99
	MR-Egger	279	1.004(0.905-1.114)	0.940	1.23E-21			
	Weighted median	279	0.977(0.898-1.063)	0.589				
	MR-PRESSO (raw, 3outliers)	276	0.966(0.962-0.969)	0.276			<1E-04	
HOA	IVW	280	0.972(0.903-1.047)	0.455	5.42E-12	0.91		0.95
	MR-Egger	280	0.978(0.868-1.101)	0.711	4.16E-12			
	Weighted median	280	1.015(0.916-1.124)	0.777				
	MR-PRESSO (raw, 1outliers)	279	0.988(0.984-0.993)	0.752			<1E-04	

Abbreviations: APOA1, Apolipoprotein A1; OA, Osteoarthritis; KHOA, OA of the hip or knee; KOA, Knee OA; HOA, Hip OA; MR, mendelian randomization; IVW, multiplicative random-effects inverse variance-weighted; SNP, single-nucleotide polymorphism; OR, odds ratio; CI, confidence interval.

Supplemental Table S2 Associations of genetically predicted HDL with OA risks in MR analyses

Main outcome	Method	No. of SNPs	OR (95% CI)	P for association	P for heterogeneity test	P for MR-Egger intercept	P for MR-PRESSO Global test	Statistical power
KHOA	IVW	333	1.004(0.954-1.057)	0.873	4.78E-34	0.18		1.00
	MR-Egger	333	1.046(0.967-1.131)	0.264	9.87E-34			
	Weighted median	333	1.051(0.987-1.120)	0.122				
	MR-PRESSO (raw,7outliers)	326	1.013(1.010-1.015)	0.604			<1E-04	
KOA	IVW	332	0.997(0.936-1.062)	0.930	8.59E-36	0.05		1.00
	MR-Egger	332	1.073(0.975-1.181)	0.152	7.02E-35			
	Weighted median	332	1.041(0.962-1.125)	0.318				
	MR-PRESSO (raw, 5outliers)	327	1.004(1.001-1.008)	0.884			<1E-04	
HOA	IVW	333	1.008(0.944-1.078)	0.805	1.94E-11	0.97		0.97
	MR-Egger	333	1.007(0.910-1.115)	0.892	1.52E-11			
	Weighted median	333	1.046(0.954-1.148)	0.338				
	MR-PRESSO (raw, 5outliers)	328	1.012(1.008-1.015)	0.711			<1E-04	

Abbreviations: HDL, high density lipoprotein cholesterol; OA, Osteoarthritis; KHOA, OA of the hip or knee; KOA, Knee OA; HOA, Hip OA; MR, mendelian randomization; IVW, multiplicative random-effects inverse variance-weighted; SNP, single-nucleotide polymorphism; OR, odds ratio; CI, confidence interval.

Supplemental Table S3 Associations of genetically predicted TG with OA risks in MR analyses

Main outcome	Method	No. of SNPs	OR (95% CI)	P for association	P for heterogeneity test	P for MR-Egger intercept	P for MR-PRESSO Global test	Statistical power
KHOA	IVW	294	0.973(0.921-1.029)	0.337	2.30E-32	0.16		1.00
	MR-Egger	294	0.930(0.855-1.012)	0.092	5.54E-32			
	Weighted median	294	0.955(0.894-1.020)	0.174				
	MR-PRESSO (raw, 9outliers)	285	0.973(0.970-0.976)	0.271			<1E-04	
KOA	IVW	294	0.989(0.925-1.057)	0.741	1.79E-30	0.07		1.00
	MR-Egger	294	0.919(0.831-1.018)	0.106	1.04E-29			
	Weighted median	294	0.998(0.922-1.079)	0.953				
	MR-PRESSO (raw, 3outliers)	292	0.946(0.942-0.950)	0.108			<1E-04	
HOA	IVW	295	0.942(0.875-1.014)	0.111	1.80E-13	0.98		0.95
	MR-Egger	295	0.941(0.841-1.053)	0.288	1.36E-13			
	Weighted median	295	0.958(0.867-1.058)	0.396				
	MR-PRESSO (raw, 3outliers)	292	0.946(0.942-0.950)	0.108			<1E-04	

Abbreviations: TG, triglycerides; OA, Osteoarthritis; KHOA, OA of the hip or knee; KOA, Knee OA; HOA, Hip OA; MR, mendelian randomization; IVW, multiplicative random-effects inverse variance-weighted; SNP, single-nucleotide polymorphism; OR, odds ratio; CI, confidence interval.

Supplemental Table S4 Associations of genetically predicted OA with lipids in MR analyses

Exposure	Main outcome	Method	No. of SNPs	OR (95% CI)	P for association	P for heterogeneity test	P for MR-Egger intercept
		IVW	22	0.981(0.935-1.030)	0.450	1.81E-24	0.345
	APOA1	MR-Egger	22	1.107(0.863-1.418)	0.433	1.65E-23	
		Weighted median	22	0.977(0.946-1.008)	0.141		
		IVW	22	0.974(0.925-1.026)	0.319	1.34E-24	0.869
	APOB	MR-Egger	22	0.996(0.762-1.303)	0.978	5.05E-25	
		Weighted median	22	0.997(0.965-1.029)	0.843		
		IVW	22	0.985(0.935-1.038)	0.575	1.50E-31	0.309
KHOA	HDL	MR-Egger	22	1.132(0.868-1.475)	0.372	5.33E-30	
		Weighted median	22	0.984(0.954-1.015)	0.307		
		IVW	22	0.974(0.922-1.029)	0.348	1.07E-28	0.802
	LDL	MR-Egger	22	1.010(0.759-1.345)	0.946	4.50E-29	
		Weighted median	22	0.995(0.964-1.028)	0.782		
		IVW	22	0.982(0.937-1.028)	0.438	1.32E-20	0.789
	TG	MR-Egger	22	1.014(0.797-1.291)	0.909	6.04E-21	
		Weighted median	22	0.999(0.968-1.032)	0.962		
		IVW	7	0.978(0.925-1.034)	0.430	4.56E-06	0.198
	APOA1	MR-Egger	7	1.206(0.910-1.599)	0.248	1.99E-4	
		Weighted median	7	0.969(0.931-1.009)	0.123		
		IVW	7	1.003(0.939-1.071)	0.929	8.18E-08	0.149
KOA	APOB	MR-Egger	7	1.318(0.958-1.813)	0.150	4.24E-05	
		Weighted median	7	1.012(0.969-1.057)	0.594		
		IVW	7	0.999(0.947-1.054)	0.968	6.36E-06	0.107
	HDL	MR-Egger	7	1.271(0.995-1.622)	0.113	0.002	
		Weighted median	7	0.986(0.947-1.026)	0.487		

		IVW	7	1.003(0.939-1.071)	0.936	9.13E-08	0.128
	LDL	MR-Egger	7	1.333(0.976-1.819)	0.130	8.26E-05	
		Weighted median	7	0.995(0.954-1.045)	0.946		
		IVW	7	0.998(0.938-1.061)	0.938	1.56E-07	0.508
	TG	MR-Egger	7	1.134(0.792-1.626)	0.523	3.05E-07	
		Weighted median	7	0.999(0.963-1.037)	0.975		
		IVW	23	0.991(0.970-1.012)	0.394	2.22E-11	0.270
	APOA1	MR-Egger	23	0.949(0.879-1.025)	0.199	9.47E-11	
		Weighted median	23	0.986(0.970-1.002)	0.089		
		IVW	23	0.999(0.962-1.037)	0.953	1.36E-43	0.354
	APOB	MR-Egger	23	0.938(0.820-1.074)	0.364	5.88E-42	
		Weighted median	23	1.000(0.983-1.017)	0.971		
		IVW	23	0.993(0.970-1.017)	0.571	6.78E-16	0.154
HOA	HDL	MR-Egger	23	0.936(0.862-1.016)	0.128	3.51E-14	
		Weighted median	23	0.981(0.966-0.997)	0.022		
		IVW	23	0.999(0.961-1.038)	0.941	6.63E-47	0.281
	LDL	MR-Egger	23	0.926(0.806-1.064)	0.291	2.44E-44	
		Weighted median	23	0.996(0.980-1.013)	0.667		
		IVW	23	0.997(0.973-1.023)	0.837	2.62E-17	0.430
	TG	MR-Egger	23	1.034(0.944-1.133)	0.481	5.43E-17	
		Weighted median	23	1.008(0.991-1.026)	0.346		

Abbreviations: APOA1, Apolipoprotein A1; APOB, Apolipoprotein B; HDL, high density lipoprotein cholesterol; LDL, low density lipoprotein cholesterol; TG, triglycerides; SNP, single-nucleotide polymorphism; OA, Osteoarthritis; KHOA, OA of the hip or knee; KOA, Knee OA; HOA, Hip OA; MR, mendelian randomization; IVW, multiplicative random-effects inverse variance-weighted; OR, odds ratio; CI, confidence interval.

Supplemental Figure S1. Diagram of Mendelian randomization framework in the current paper.

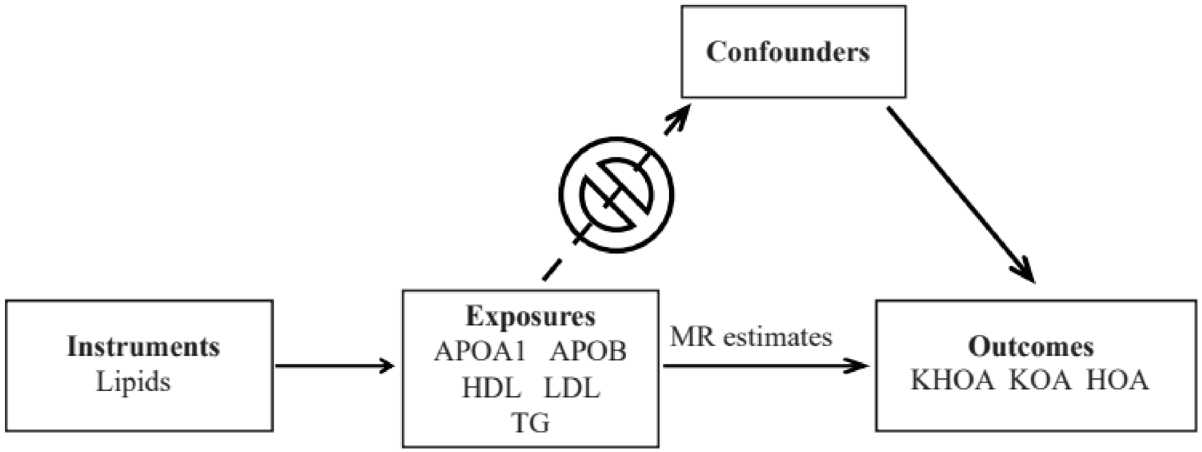

Abbreviations: APOA1, Apolipoprotein A1; APOB, Apolipoprotein B; HDL, high density lipoprotein cholesterol; LDL, low density lipoprotein cholesterol; TG, triglycerides; MR, mendelian randomization; KHOA, OA of the hip or knee; KOA, Knee OA; HOA, Hip OA

Supplemental Figure S2. Forest plots of variant specific inverse variance estimates for causal association between lipids and OA.

1.APOA1 and OA

KOA

HOA

KHOA

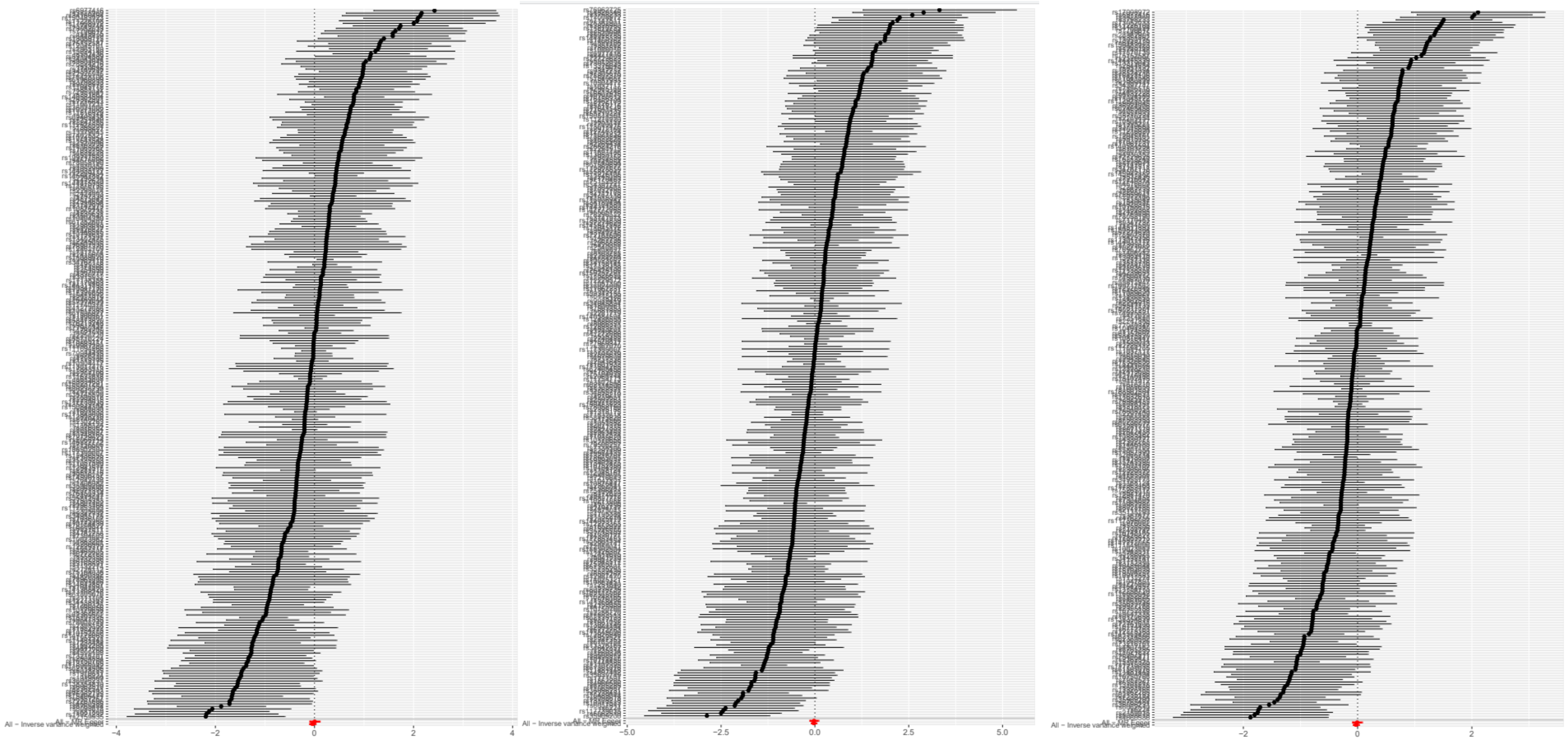

Abbreviations: APOA1, Apolipoprotein A1; OA, Osteoarthritis; KHOA, OA of the hip or knee; KOA, Knee Osteoarthritis; HOA, Hip Osteoarthritis.

2.APOB and OA

KOA | HOA | KHOA

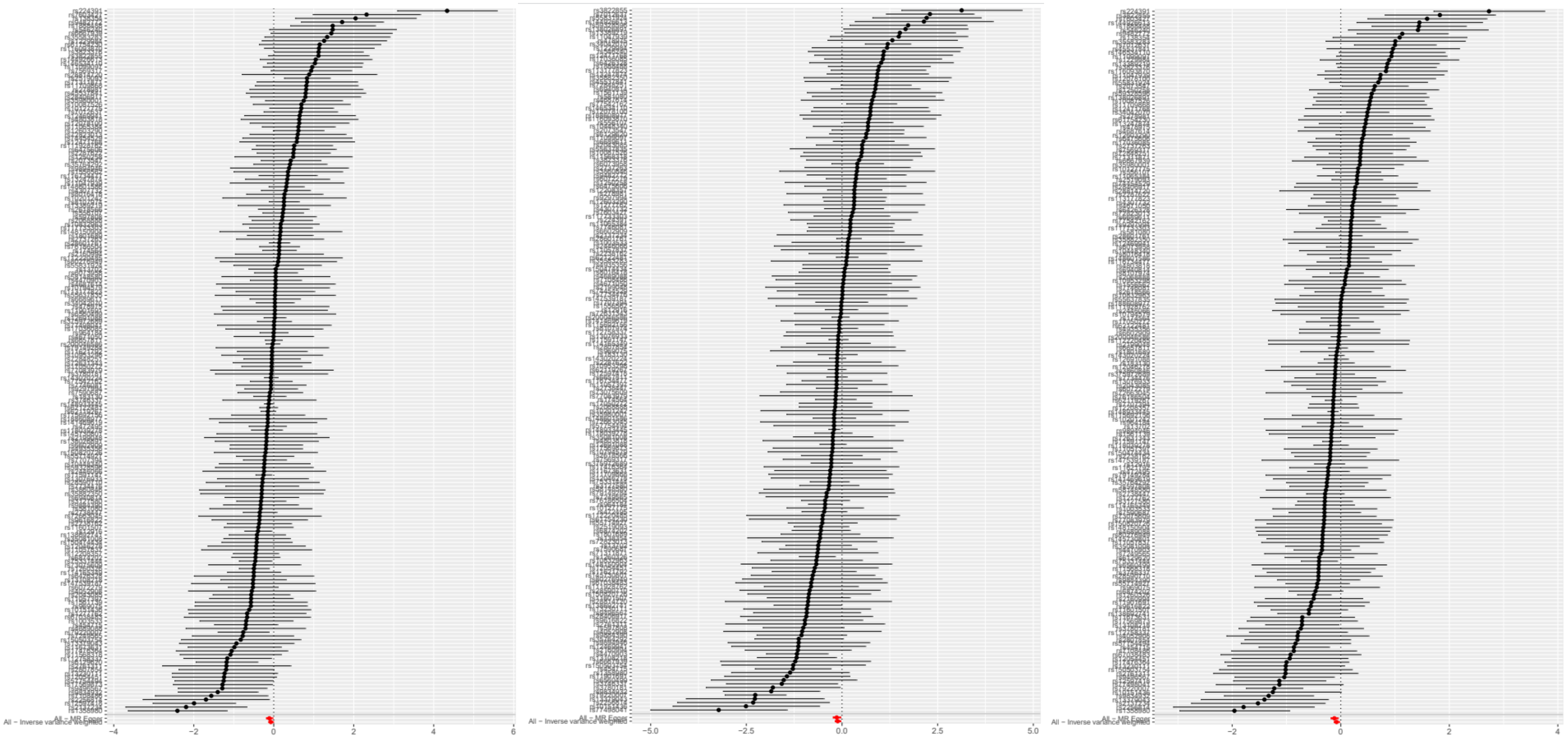

Abbreviations: APOB, Apolipoprotein B; OA, Osteoarthritis; KHOA, OA of the hip or knee; KOA, Knee Osteoarthritis; HOA, Hip Osteoarthritis.

3.HDL and OA

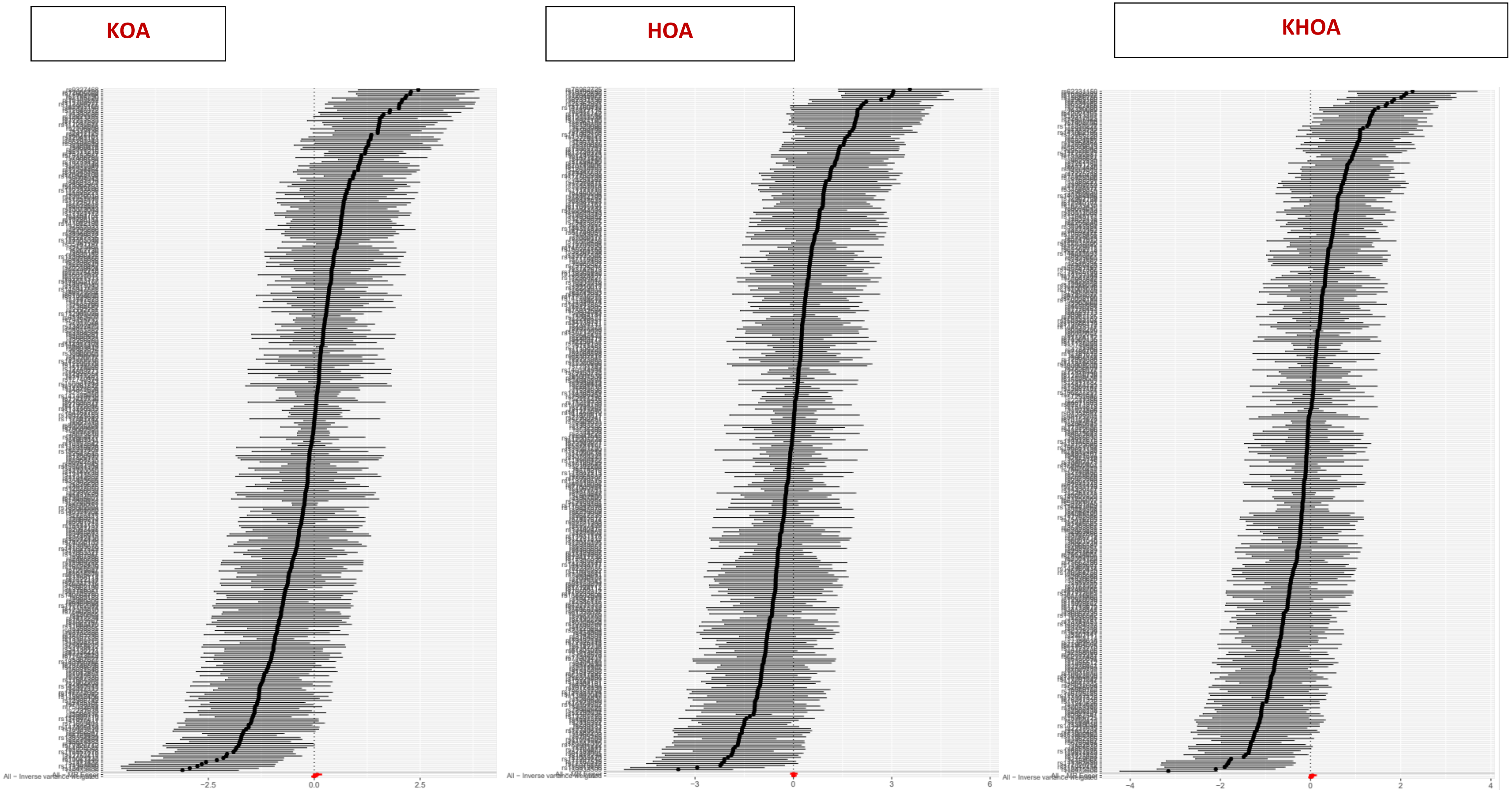

Abbreviations: HDL, high density lipoprotein cholesterol; OA, Osteoarthritis; KHOA, OA of the hip or knee; KOA, Knee Osteoarthritis; HOA, Hip Osteoarthritis.

4.LDL and OA

KOA

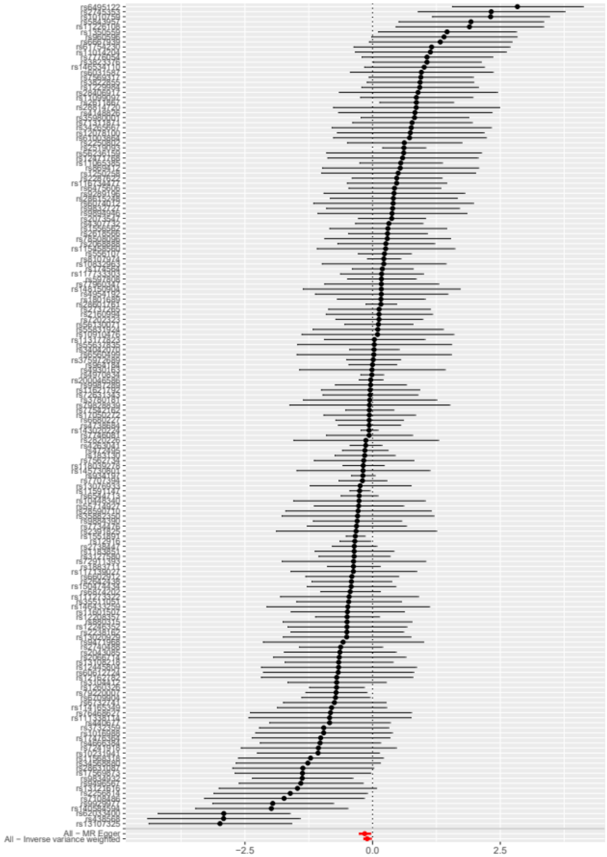

HOA

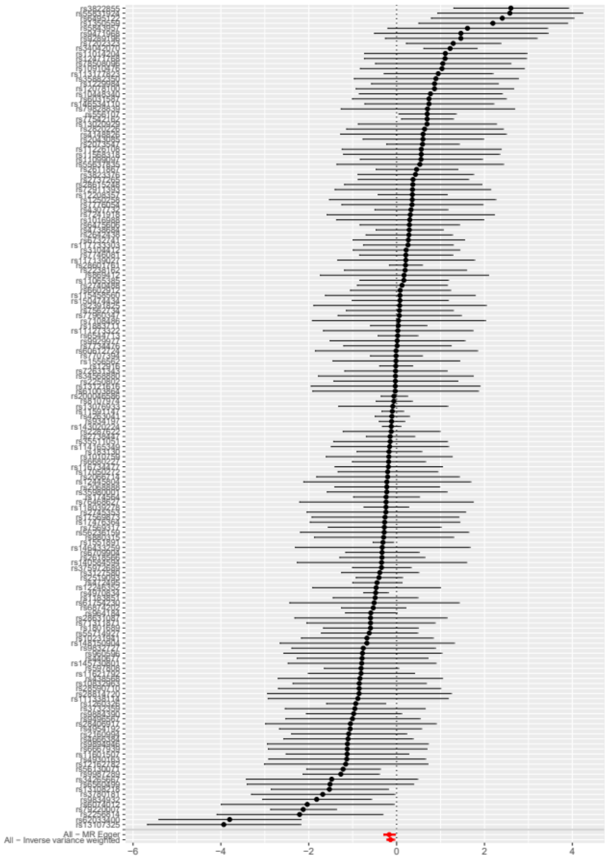

KHOA

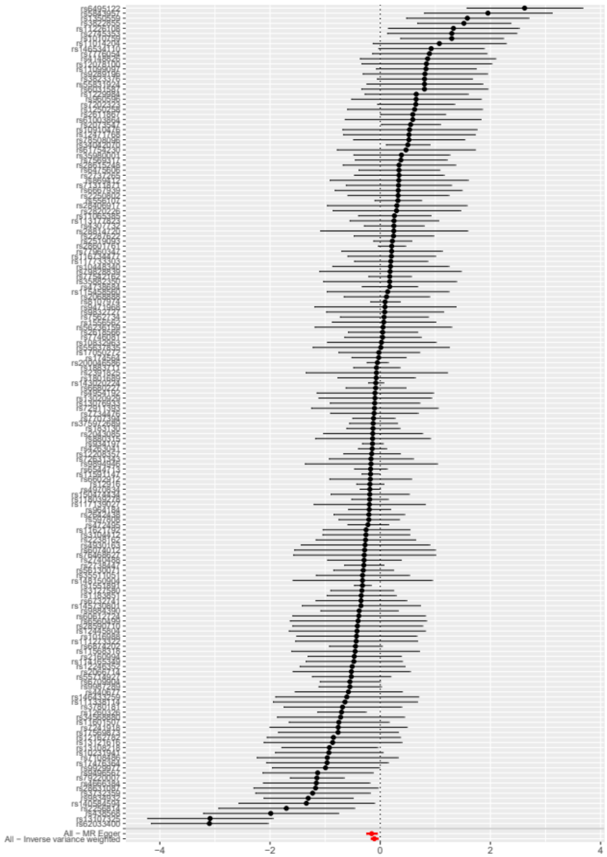

Abbreviations: LDL, low density lipoprotein cholesterol; OA, Osteoarthritis; KHOA, OA of the hip or knee; KOA, Knee Osteoarthritis; HOA, Hip Osteoarthritis.

5.TG and OA

KOA

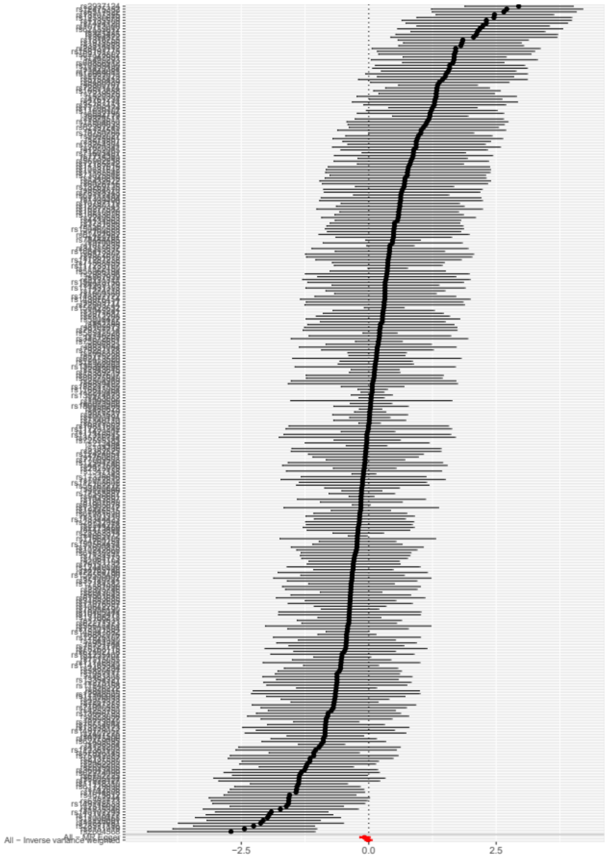

HOA

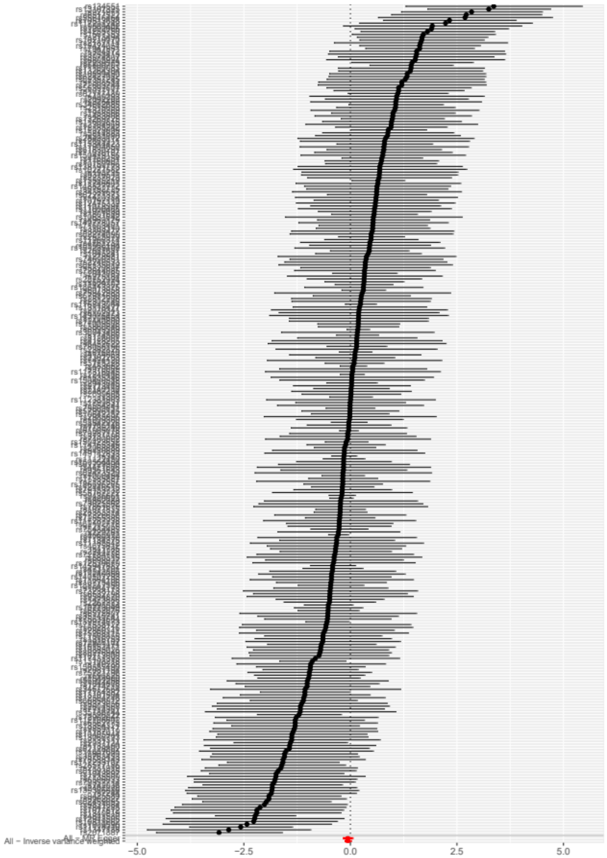

KHOA

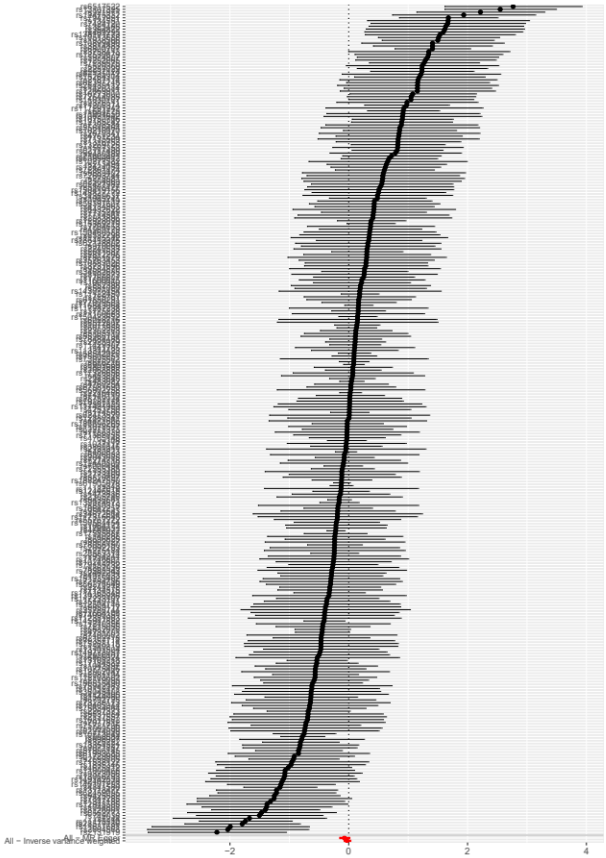

Abbreviations: TG, triglycerides; OA, Osteoarthritis; KHOA, OA of the hip or knee; KOA, Knee Osteoarthritis; HOA, Hip Osteoarthritis.

Supplemental Figure S3. Funnel plots of causal association between lipids and OA.

1.APOA1 and OA

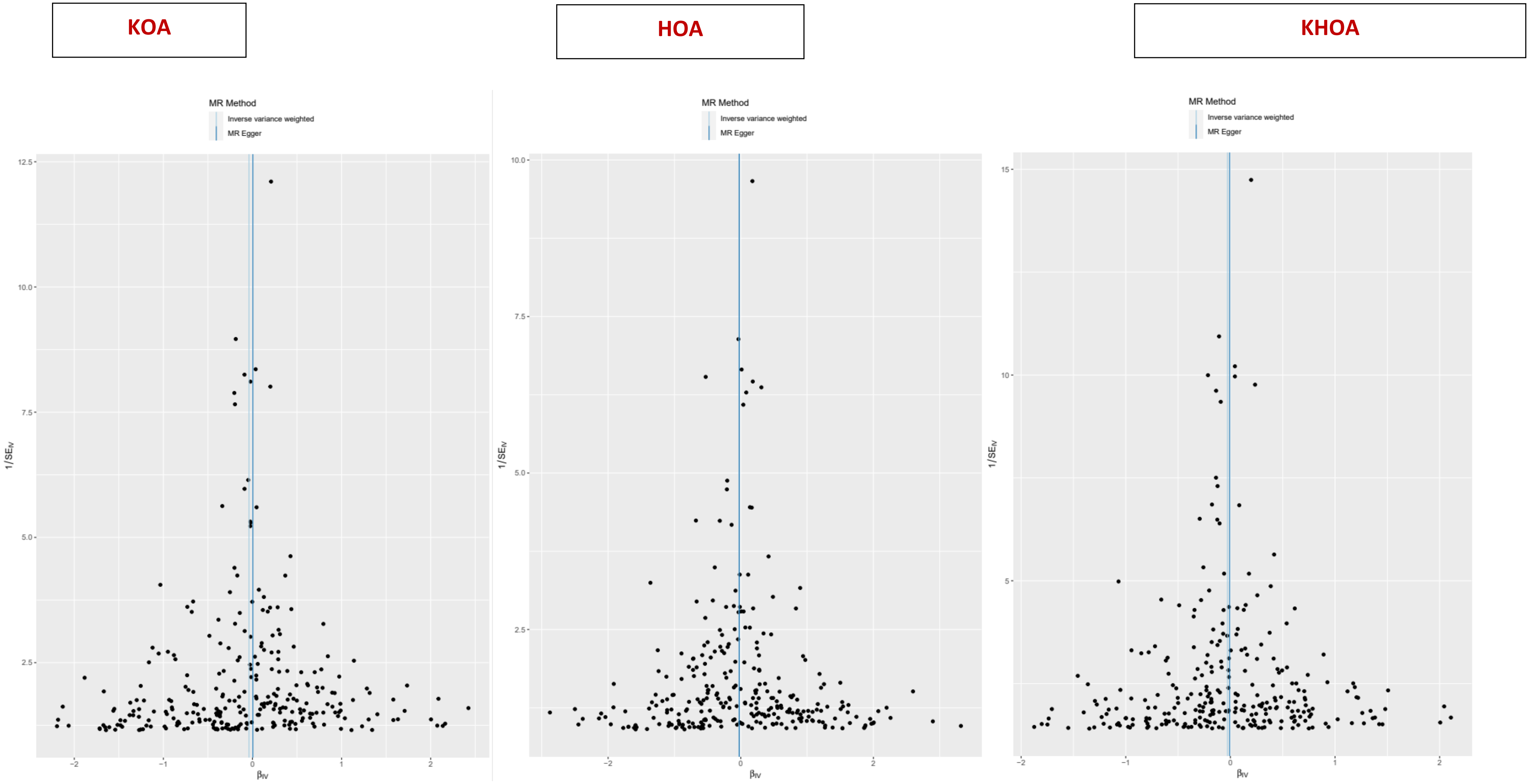

Abbreviations: APOA1, Apolipoprotein A1; OA, Osteoarthritis; KHOA, OA of the hip or knee; KOA, Knee Osteoarthritis; HOA, Hip Osteoarthritis.

2.APOB and OA

KOA

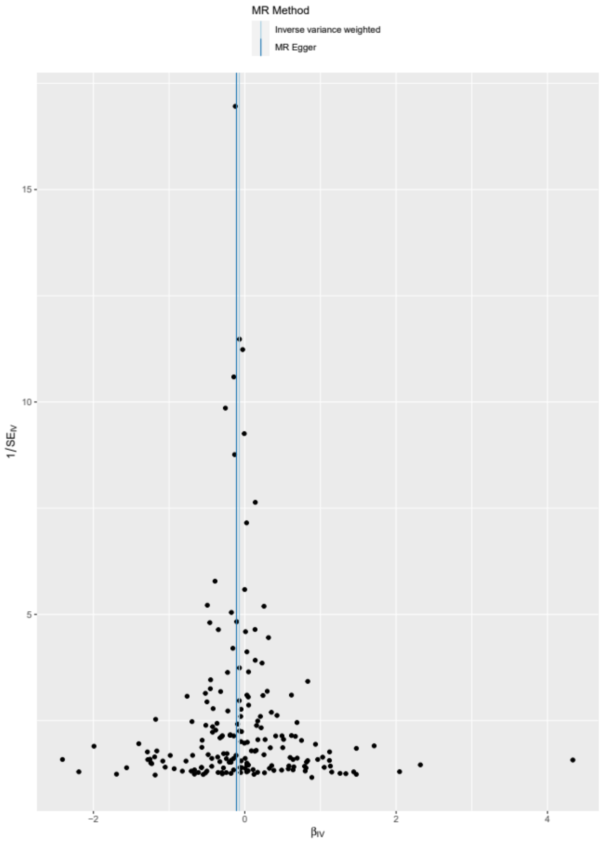

HOA

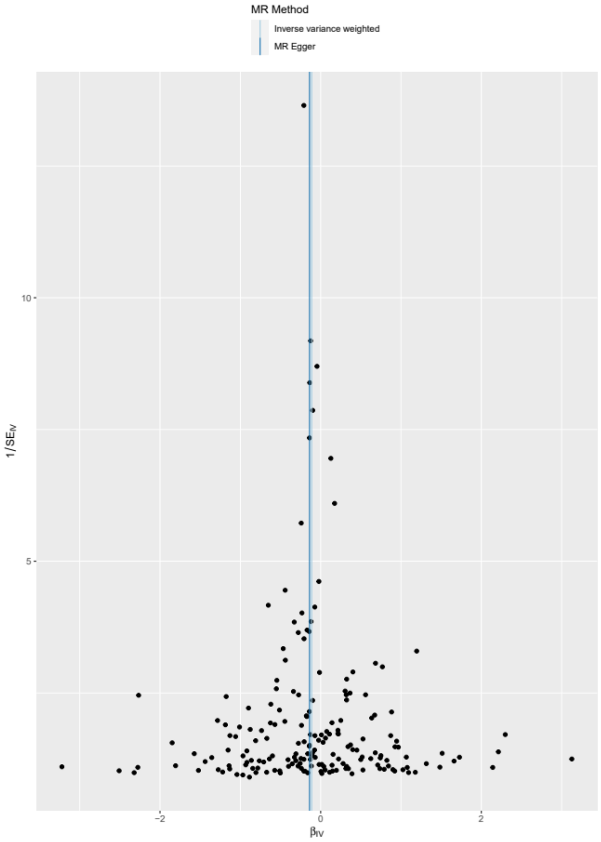

KHOA

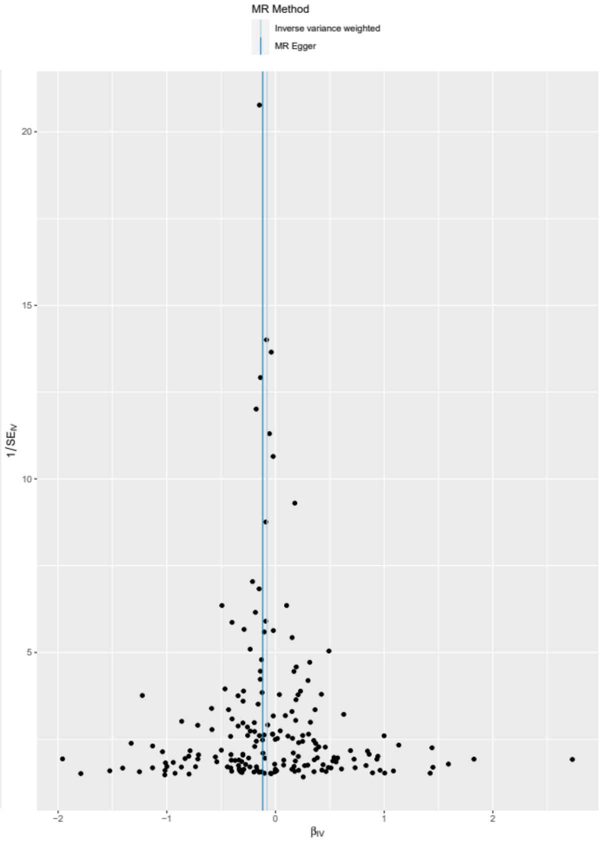

Abbreviations: APOB, Apolipoprotein B; OA, Osteoarthritis; KHOA, OA of the hip or knee; KOA, Knee Osteoarthritis; HOA, Hip Osteoarthritis.

3.HDL and OA

KOA

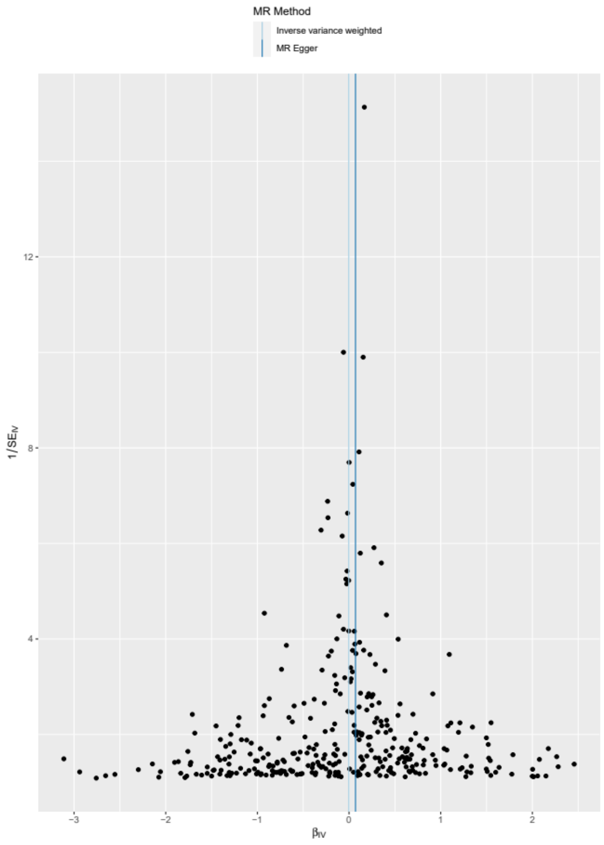

HOA

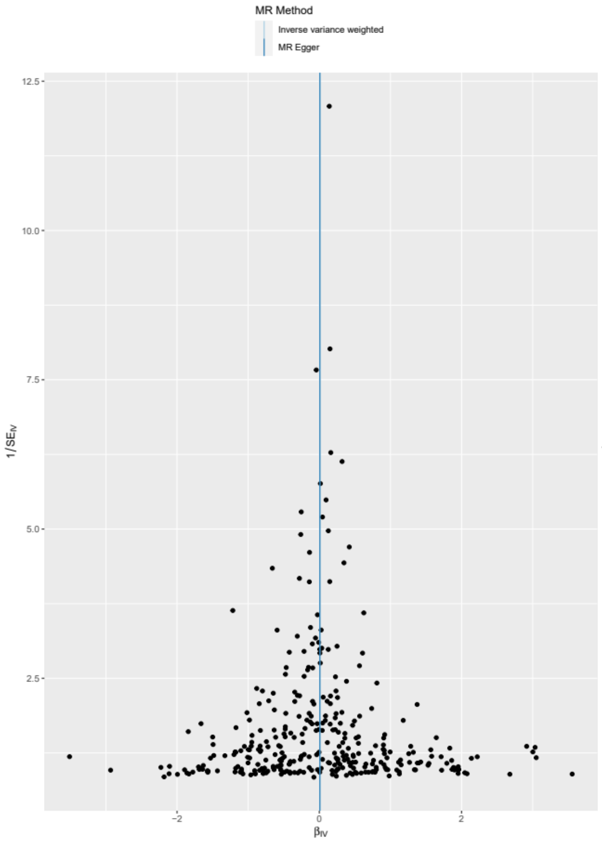

KHOA

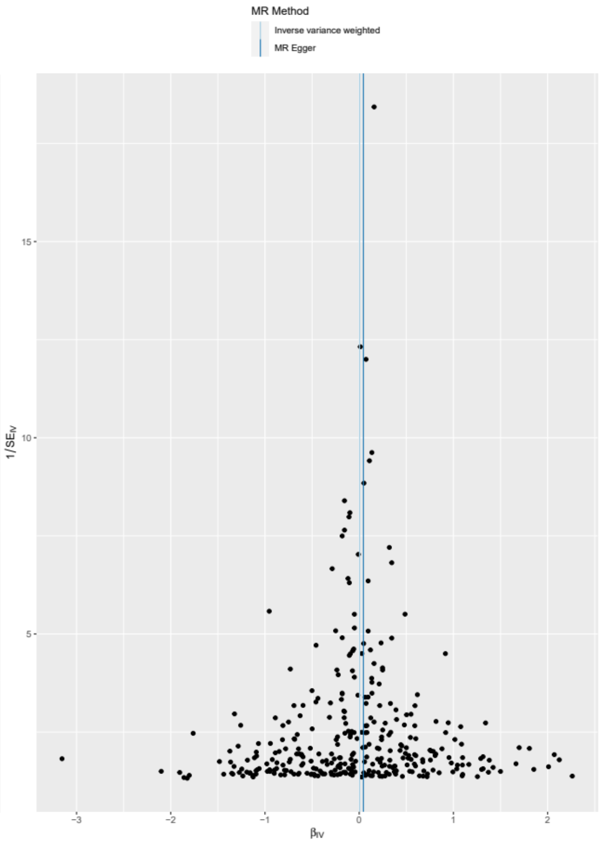

Abbreviations: HDL, high density lipoprotein cholesterol; OA, Osteoarthritis; KHOA, OA of the hip or knee; KOA, Knee Osteoarthritis; HOA, Hip Osteoarthritis.

4.LDL and OA

KOA

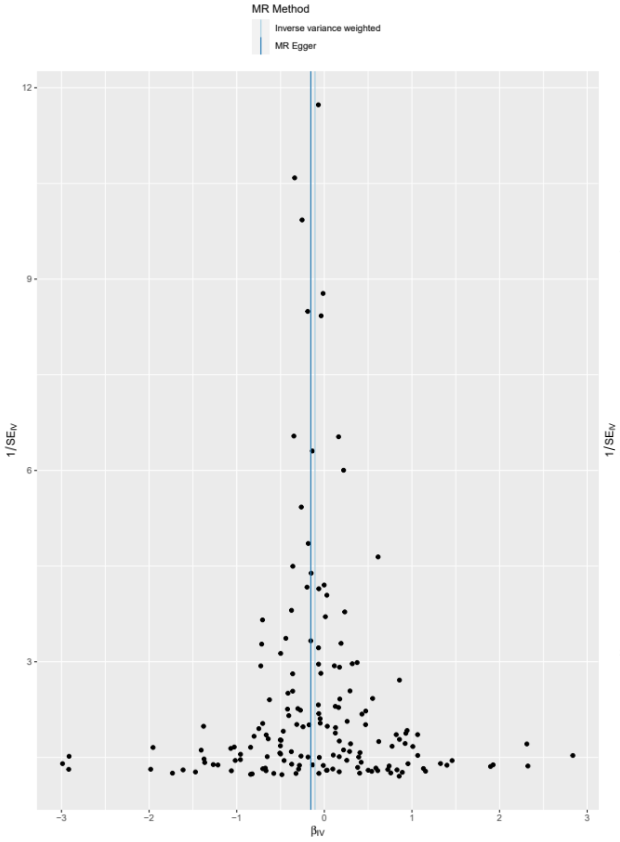

HOA

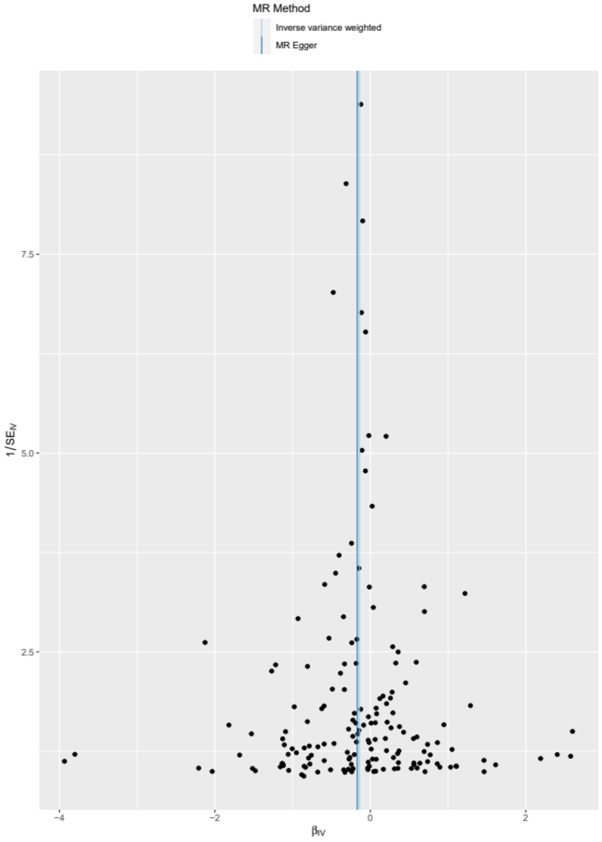

KHOA

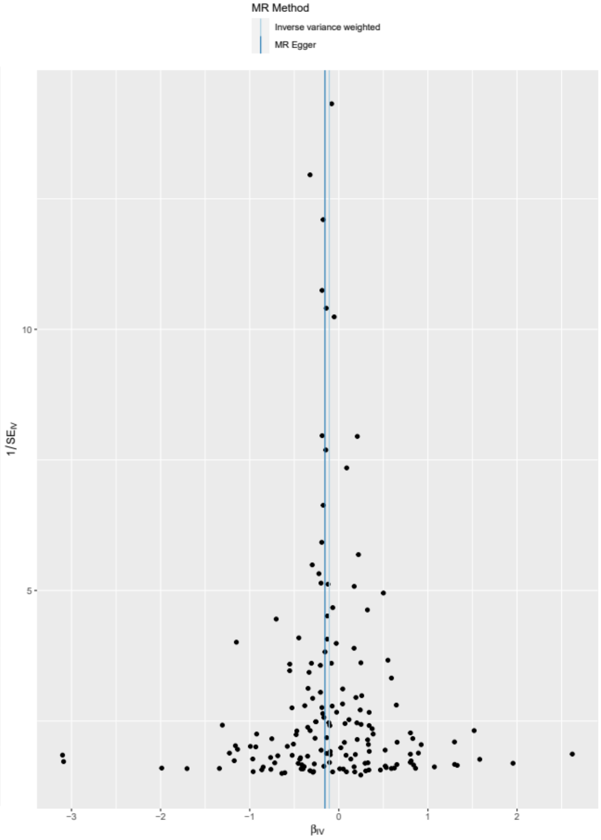

Abbreviations: LDL, low density lipoprotein cholesterol; OA, Osteoarthritis; KHOA, OA of the hip or knee; KOA, Knee Osteoarthritis; HOA, Hip Osteoarthritis.

5.TG and OA

KOA

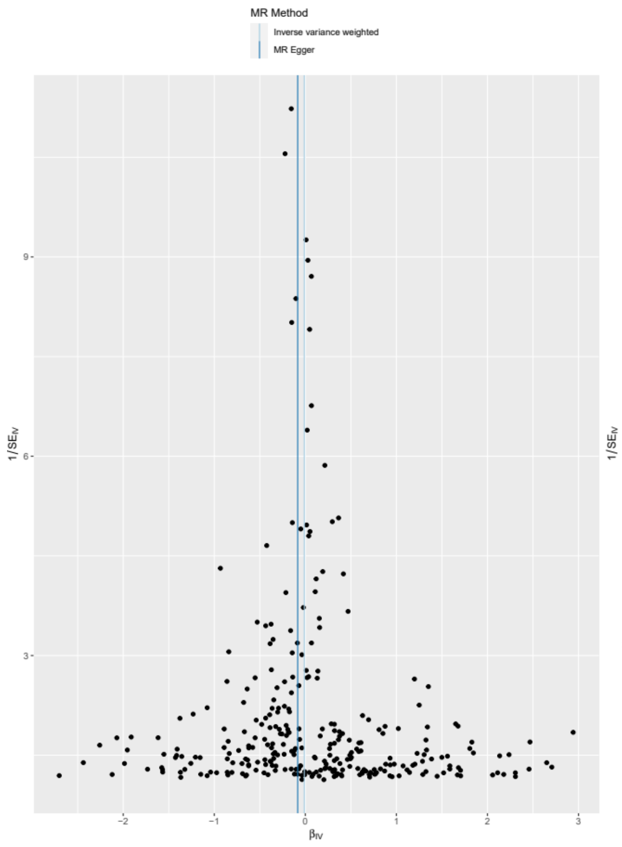

HOA

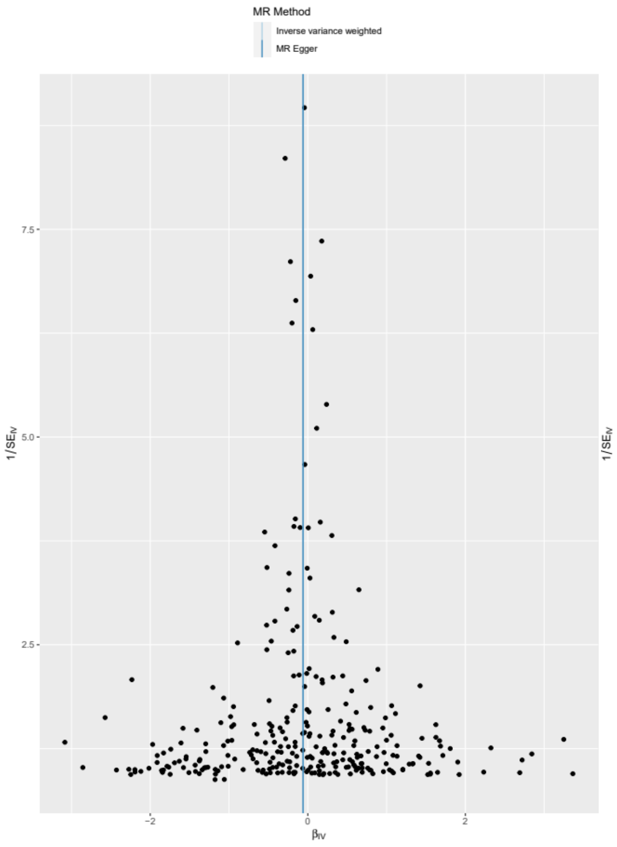

KHOA

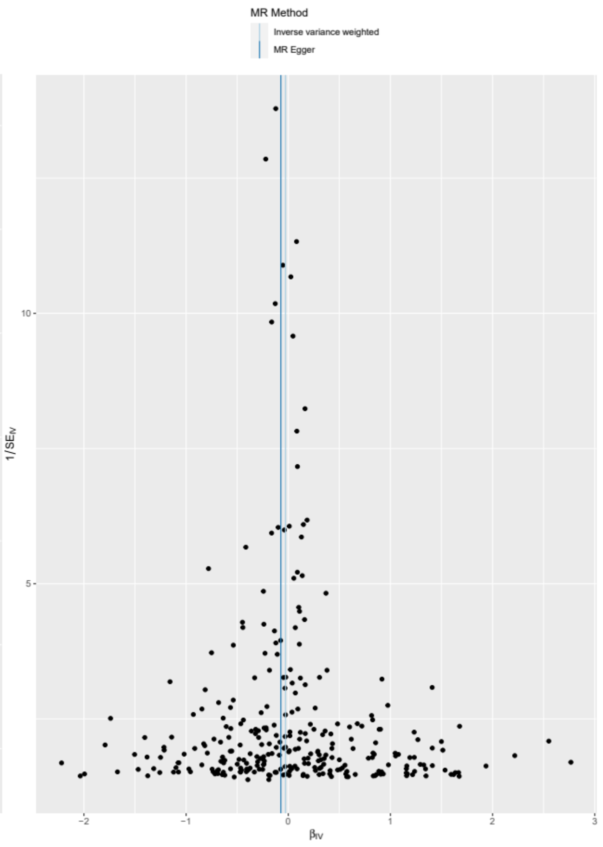

Abbreviations: TG, triglycerides; OA, Osteoarthritis; KHOA, OA of the hip or knee; KOA, Knee Osteoarthritis; HOA, Hip Osteoarthritis.

Supplemental Figure S4. Leave-one-out plots to assess if a single variant is driving the association between lipids and OA.

1.APOA1 and OA

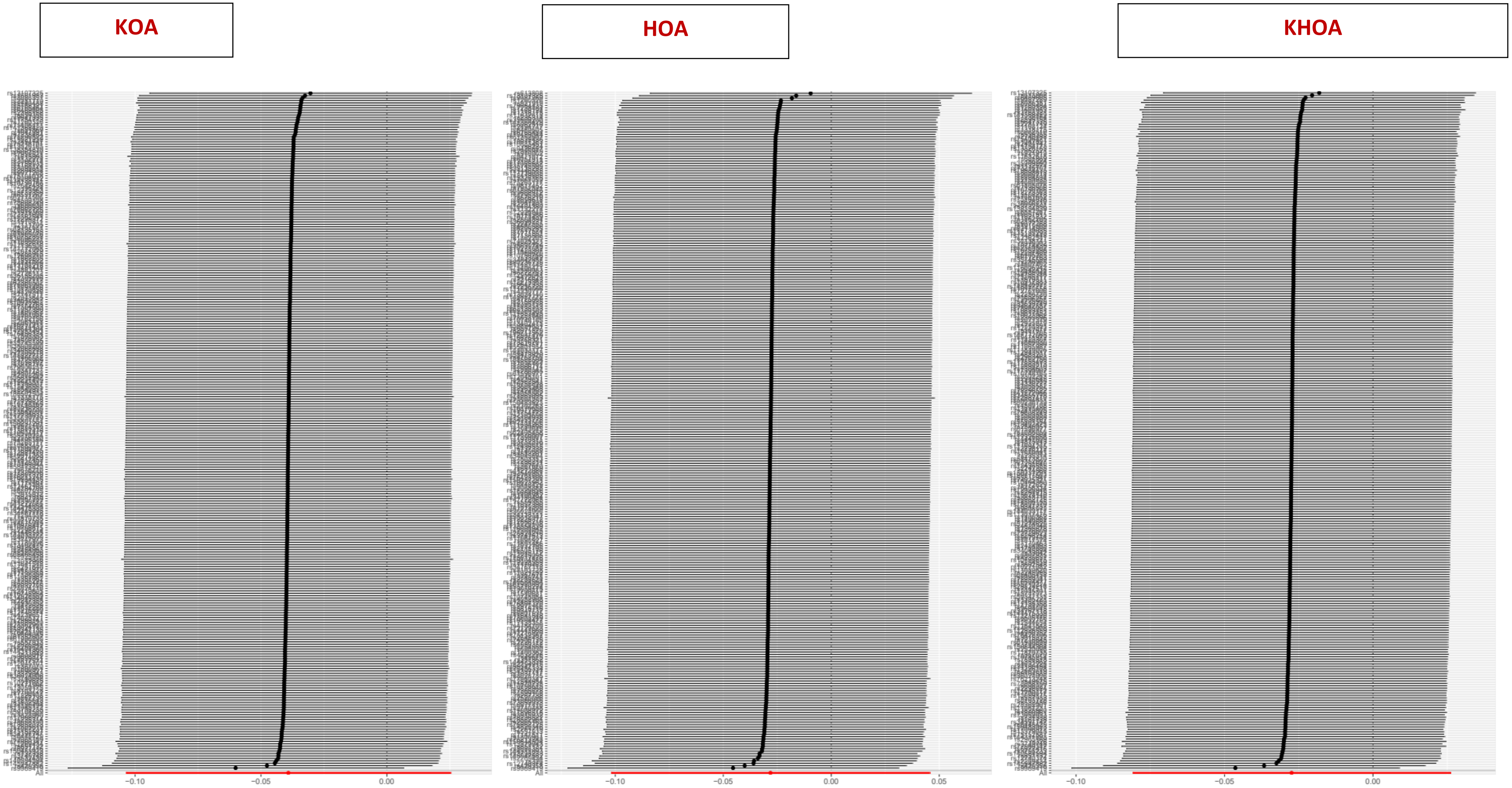

Abbreviations: APOA1, Apolipoprotein A1; OA, Osteoarthritis; KHOA, OA of the hip or knee; KOA, Knee Osteoarthritis; HOA, Hip Osteoarthritis.

2.APOB and OA

KOA HOA KHOA

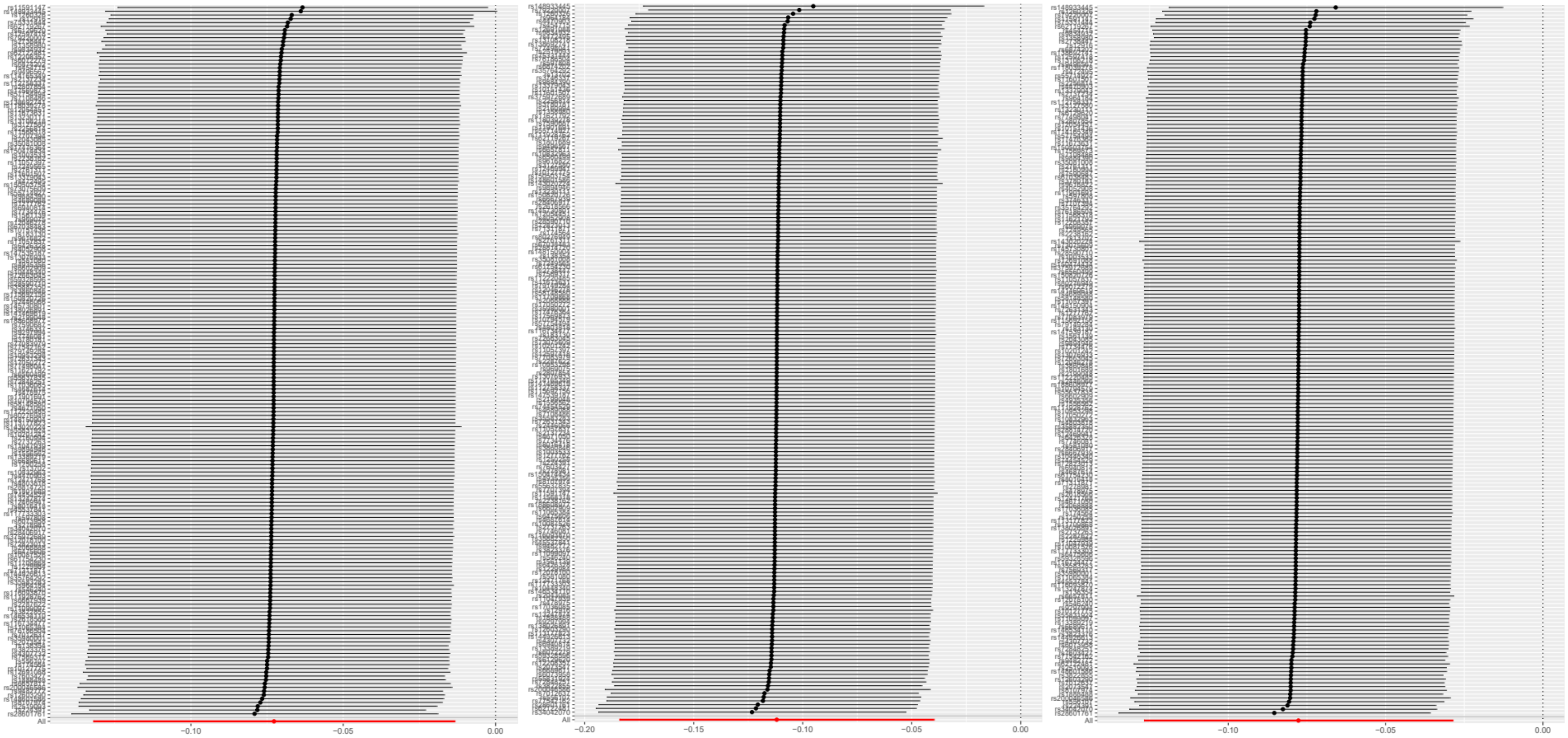

Abbreviations: APOB, Apolipoprotein B; OA, Osteoarthritis; KHOA, OA of the hip or knee; KOA, Knee Osteoarthritis; HOA, Hip Osteoarthritis.

3.HDL and OA

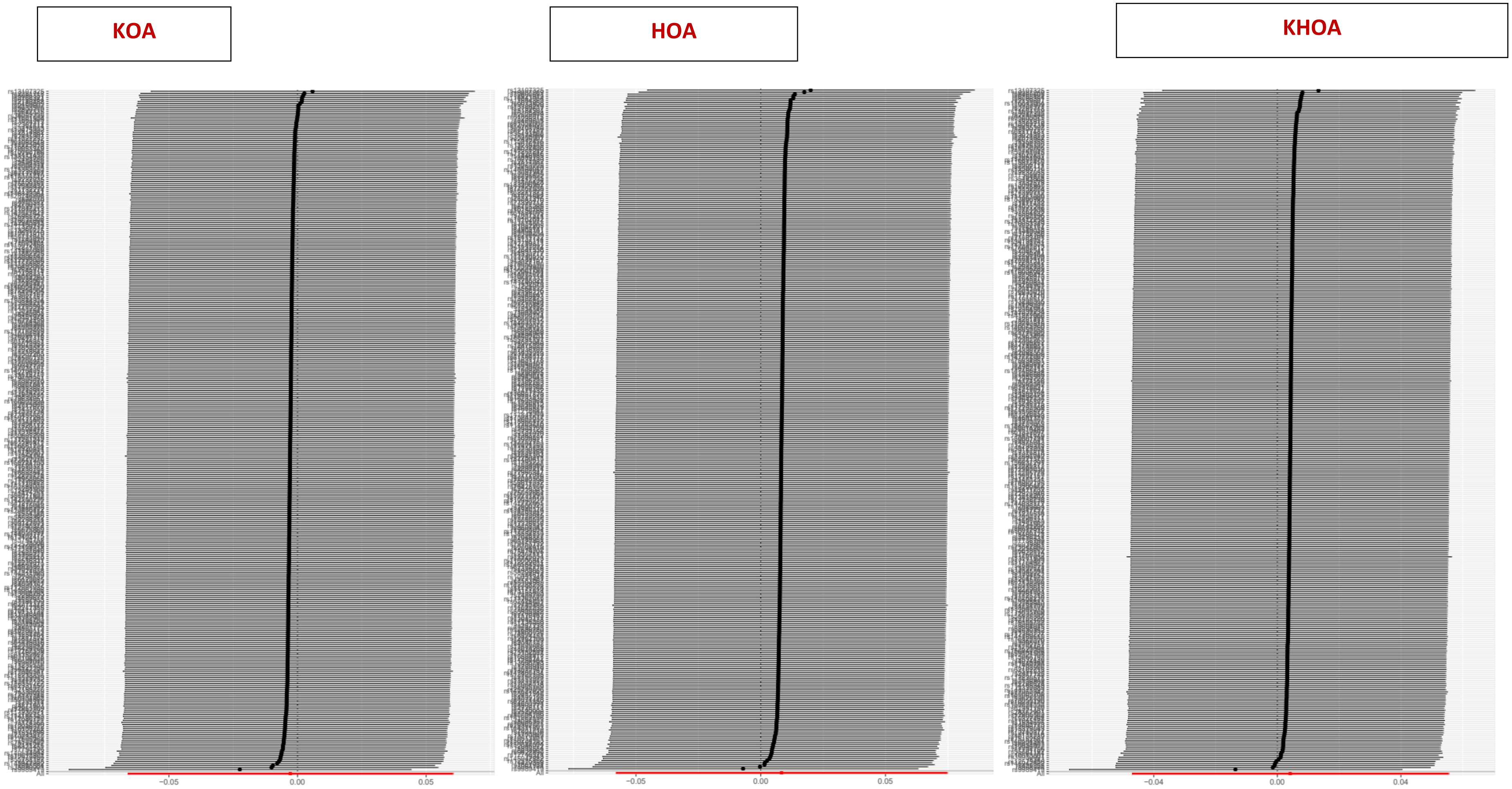

Abbreviations: HDL, high density lipoprotein cholesterol; OA, Osteoarthritis; KHOA, OA of the hip or knee; KOA, Knee Osteoarthritis; HOA, Hip Osteoarthritis.

4.LDL and OA

KOA HOA KHOA

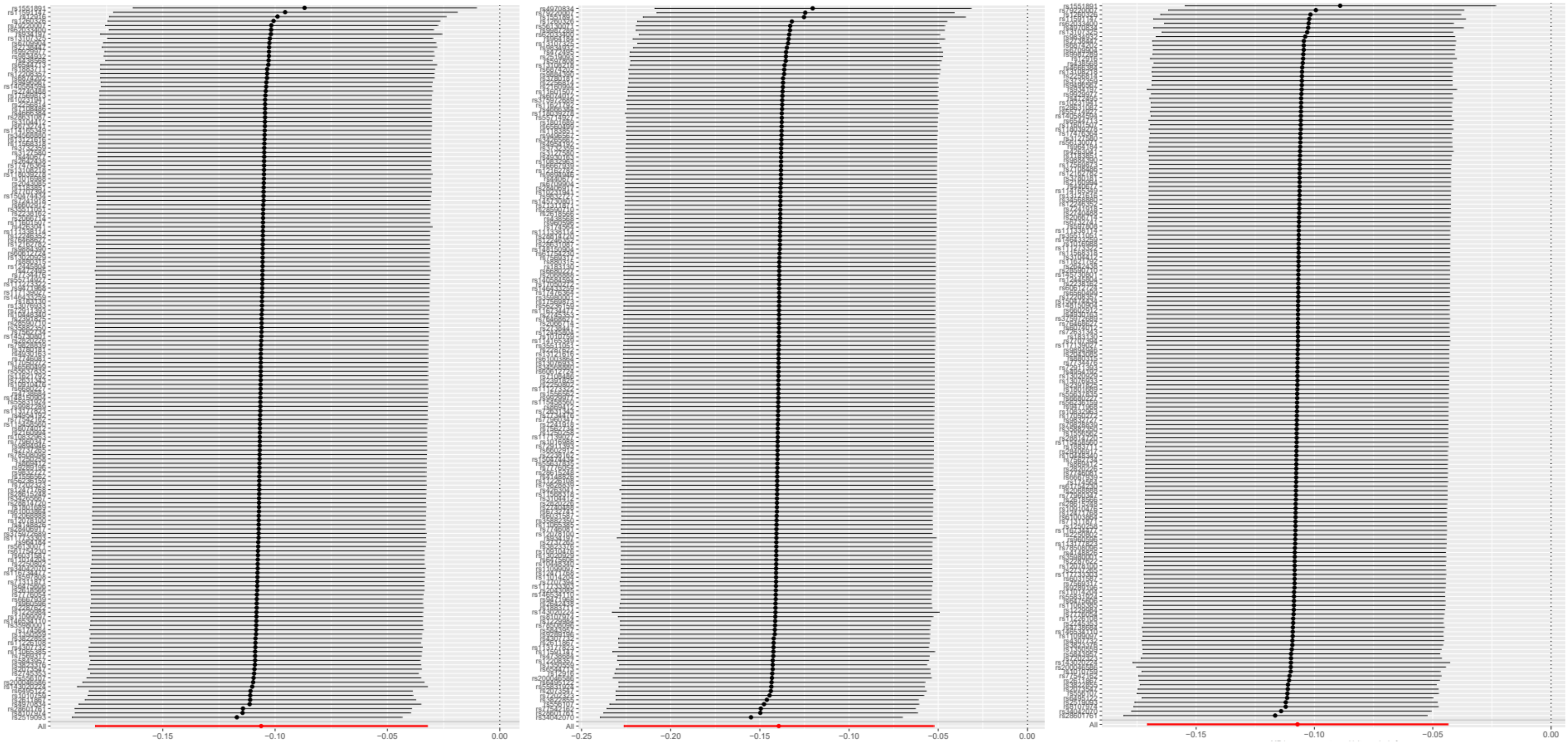

Abbreviations: LDL, low density lipoprotein cholesterol; OA, Osteoarthritis; KHOA, OA of the hip or knee; KOA, Knee Osteoarthritis; HOA, Hip Osteoarthritis.

5.TG and OA

KOA HOA KHOA

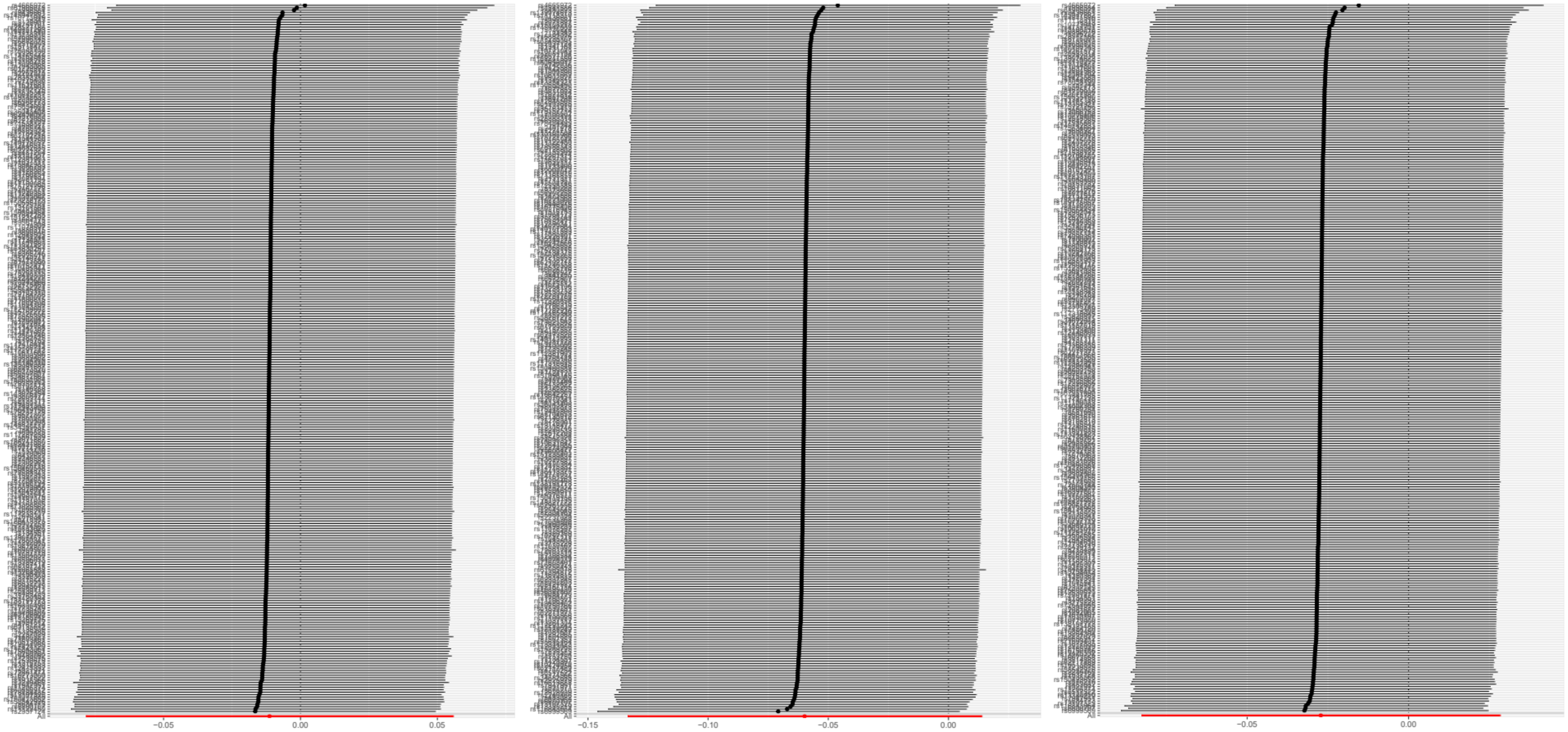

Abbreviations: TG, triglycerides; OA, Osteoarthritis; KHOA, OA of the hip or knee; KOA, Knee Osteoarthritis; HOA, Hip Osteoarthritis.
